# Assessing treatment fidelity and contamination in a cluster randomised controlled trial of motivational interviewing and cognitive behavioural therapy skills in type 2 diabetes

**DOI:** 10.1186/s12875-018-0742-5

**Published:** 2018-05-10

**Authors:** Nicholas Magill, Helen Graves, Nicole de Zoysa, Kirsty Winkley, Stephanie Amiel, Emma Shuttlewood, Sabine Landau, Khalida Ismail

**Affiliations:** 1 Department of Biostatistics and Health Informatics, Institute of Psychiatry, Psychology and Neuroscience, King’s College London, 16 De Crespigny Park, London, SE5 8AF UK; 2 Department of Psychological Medicine, Institute of Psychiatry, Psychology and Neuroscience, King’s College London, London, UK; 3 Diabetes and Nutritional Sciences Division, School of Medicine, King’s College London, London, UK; 4 Institute of Diabetes, Endocrinology and Obesity, King’s Health Partners, London, UK

**Keywords:** Treatment contamination, Randomised controlled trial, Diabetes, Self-management

## Abstract

**Background:**

Competencies in psychological techniques delivered by primary care nurses to support diabetes self-management were compared between the intervention and control arms of a cluster randomised controlled trial as part of a process evaluation. The trial was pragmatic and designed to assess effectiveness. This article addresses the question of whether the care that was delivered in the intervention and control trial arms represented high fidelity treatment and attention control, respectively.

**Methods:**

Twenty-three primary care nurses were either trained in motivational interviewing (MI) and cognitive behavioural therapy (CBT) skills or delivered attention control. Nurses’ skills in these treatments were evaluated soon after training (treatment arm) and treatment fidelity was assessed after treatment delivery for sessions midway through regimen (both arms) using the Motivational Interviewing Treatment Integrity (MITI) domains and Behaviour Change Counselling Index (BECCI) based on consultations with 151 participants (45% of those who entered the study). The MITI Global Spirit subscale measured demonstration of MI principles: evocation, collaboration, autonomy/support.

**Results:**

After training, median MITI MI-Adherence was 86.2% (IQR 76.9–100%) and mean MITI Empathy was 4.09 (SD 1.04). During delivery of treatment, in the intervention arm mean MITI Spirit was 4.03 (SD 1.05), mean Empathy was 4.23 (SD 0.89), and median Percentage Complex Reflections was 53.8% (IQR 40.0–71.4%). In the attention control arm mean Empathy was 3.40 (SD 0.98) and median Percentage Complex Reflections was 55.6% (IQR 41.9–71.4%).

**Conclusions:**

After MI and CBT skills training, detailed assessment showed that nurses had basic competencies in some psychological techniques. There appeared to be some delivery of elements of psychological treatment by nurses in the control arm. This model of training and delivery of MI and CBT skills integrated into routine nursing care to support diabetes self-management in primary care was not associated with high competency levels in all skills.

**Trial registration:**

ISRCTN75776892; date registered: 19/05/2010.

## Background

Psychological treatments are complex interventions, which are generally defined as treatments that comprise several interacting components or active ingredients [1]. In randomised controlled trials (RCTs) of such interventions, the standard intention-to-treat analysis can estimate the causal effect of treatment offer on outcome but does not shed any light on whether the two competing treatment offers were delivered to participants as intended. Process evaluations are a set of methodologies for assessing the implementation, mechanisms, and context of an intervention [2]. Process evaluation of an RCT has become increasingly important because it may help to explain why an intervention was or was not effective [3]. In trials of psychological treatments the most commonly studied process is fidelity. This is defined as the consistency of what was implemented with what was intended [4]. Related to this concept is that of clinician competency, which is a clinician’s ability to implement a technique [5]. Its assessment is particularly important in trials where treatment is delivered by a non-specialist.

Randomised controlled trials of psychological treatments are increasingly assessing clinician competency, using methods such as audio recordings, clinical notes, and random observations of delivered therapy. These are frequently done in the active intervention arm but rarely in the control condition. The implication of this is that such process evaluation in trials may be missing the problem of treatment contamination, where participants in the control group receive elements of the active intervention [6]. An evaluation of what treatment participants in the control group receive is important in trials and especially when the comparator is an attention control that can contain some active ingredients.

The context of the assessment of treatment fidelity described in this article is a trial of a psychological treatment for people who suffer from type 2 diabetes (T2D) with suboptimal glycaemic control [7]. Suboptimal control is common amongst people with T2D despite medical and educational interventions [8–10]. Reasons are multifactorial and include psychological barriers such as denial, depression, stigma, and fears around insulin [11, 12]. The need for psychological care to help motivate patients towards lifestyle adjustment has been emphasised in national guidelines [13], and psychological interventions have demonstrated promise in improving outcomes in T2D [14]. The rationale for the Diabetes 6 (D6) trial was based on the need to find cost-effective ways of competently delivering diabetes-informed psychological treatments. Emerging evidence suggests that allied healthcare professionals can be trained to provide basic psychological interventions and that this is associated with an improvement in glycaemic control in type 1 diabetes. For example, hospital diabetes nurses have been trained to deliver diabetes-specific psychological therapy competently and primary care nurses have successfully been trained to use motivational techniques to improve oral medication adherence in people with T2D [15, 16]. In addition, a study of nurse-delivered motivational interviewing (MI) in primary care showed that nurses had some basic competency but this did not develop over time [17].

The D6 study was a cluster RCT evaluating the effectiveness of an intervention combining motivational interviewing (MI) and cognitive behavioural therapy (CBT) skills compared to an attention control which did not include any psychological components. One reason for using cluster randomisation at the level of the primary care nurse was to avoid treatment contamination that was anticipated if a given nurse were asked to provide both control and active treatments. The psychological interventions were both evidence-based approaches aimed at producing behavioural change, with evidence suggesting that integrating MI and CBT may be beneficial [18]. The treatment in the control arm consisted of standard diabetes care, with primary care nurses scheduled to meet participants for the same number of times and same duration as those in the active intervention arm. Participants were offered six face-to-face sessions followed by six sessions in a format agreed with the nurse. The primary aim of the trial was to investigate the effect of psychological treatment offer on glycated haemoglobin. Recruitment criteria included evidence of suboptimal control prior to entry into the study and current receipt of standard care.

The D6 trial provided an opportunity to assess treatment fidelity, using audio recordings of treatment sessions. This enabled an examination of whether nurses could be trained to deliver psychological therapy competently to participants within the active intervention arm. It also allowed an assessment of whether participants allocated to the attention control arm received psychological therapy – that is, whether contamination occurred. This article describes the fidelity assessment of the treatments delivered to participants in the two trial arms and represents a secondary analysis.

The aims of this study were to: i) assess whether D6 nurses achieved competencies in psychological therapy delivery at the end of the training period, ii) describe differences between end of training and delivery of intervention, iii) compare the levels of receipt of psychological treatment (MI and CBT skills) between the active intervention and control arms, and iv) determine to what extent the intervention and control treatments represented high fidelity MI and CBT or standard diabetes care, respectively.

## Methods

### Setting and trial design

The trial was set within 23 primary care surgeries in south London. Large surgeries (≥6000 patients) were invited to participate if they had a nurse providing diabetes care. Interventions were allocated at the surgery level (clusters). Ethical approval was granted by the King’s College Hospital Research Ethics Committee (reference 09/H0808/97) and by the respective Primary Care Trusts (reference RDLSLBex 534 and 2010/403/W). Informed written consent was obtained from all individual participants included in the study. The trial was registered with ISRCTN (ISRCTN75776892) on 19 May 2010.

### The training programme

The training programme for nurses in the intervention arm of the RCT was developed and delivered by an experienced clinical psychologist using both didactic and practicum strategies. Nurses were trained in six MI/CBT skills: active listening, managing resistance, directing change, supporting self-efficacy, addressing health beliefs, and shaping behaviours. The initial interactive training workshops were conducted over 12 3-hourly sessions and the nurses were given a handbook for ongoing reference. The focus was on increasing patients’ motivation to improve their diabetes control and then collaboratively addressing key self-care behaviours such as medication adherence, blood sugar testing, physical activity, and dietary changes.

### Techniques taught in MI and CBT

MI is a collaborative, person-centred approach to working with people in order to elicit and strengthen their motivation and commitment to change [19]. It has been found to be more effective than traditional advice-giving in the treatment of a range of behavioural problems and diseases, including diabetes [20]. CBT has been found to be effective at improving adjustment to diagnosis and self-management of diabetes [21]. It aims to achieve this by helping people to identify and restructure unhelpful cognitions, teaching behavioural strategies, and supporting people to develop helpful coping strategies.

### Clinical supervision

Nurses in the intervention group attended monthly supervision with the trial psychologist either in person at monthly group sessions or over the telephone if they were not able to attend throughout the delivery of the intervention. E-mail support was also offered for individual cases.

### Assessment of treatment fidelity and competency

All nurses who participated in the D6 study were required by protocol to record their treatment consultations with participants digitally. A sample of recordings from nurses in the intervention arm from shortly after the end of training was used to assess competency. Another sample of recordings from both trial arms that was representative of participants’ treatment receipt was selected in order to assess fidelity.

The definition, assessment, and difficulties of addressing treatment fidelity in research studies have been extensively discussed elsewhere in the literature [22–24]. A definition that is consistently used, and will be used for the purpose of this study, is that fidelity comprises both adherence and competence [24]. Adherence refers to whether the appropriate procedures were followed for that clinical intervention whereas competence refers to whether these procedures were implemented to an adequate level.

The Motivational Interviewing Treatment Integrity (MITI) Scale, version 3.1.1 [25, 26], was utilised to measure competence and skills used in both groups of nurses. A Global Spirit score is intended to capture the overall demonstration of MI principles, and a Global Empathy score is intended to capture the extent to which the clinician understands, or attempts to understand the patient’s perspective. Further measures of clinician behaviours include the use of simple reflections, complex reflections, open questions, and closed-ended questions. Scores are also calculated for MI adherent and non-adherent counselling behaviours. The possible ranges and threshold levels for subscales (as specified by the scale’s authors) are given in Table 1.

**Table 1:** Minimums, maximums, and proficiency and competency thresholds for the MITI and BECCI scales [26, 27]

MITI summary scores	Minimum (lowest score)	Maximum (highest score)	“Beginning proficiency” thresholds	“Competency” thresholds
Global Spirit and Global Empathy	1	5	Average of 3.5	Average of 4
Reflection-to-Question Ratio	0	–	1	2
Percent Open Questions	0	100	50%	70%
Percent Complex Reflections	0	100	40%	50%
Percent MI-Adherent	0	100	90%	100%
BECCI summary score				
Practitioner Score	0	4	–	–

The Behaviour Change Counselling Index (BECCI) [27] was designed to assist trainers in assessing a clinician’s competence in using behaviour change counselling in consultations. It was included here in order to assess nurses’ competence in eliciting patients’ thoughts and cognitions, therefore addressing the CBT element of the intervention. BECCI comprises 11 items which are scored from zero to four (0 = *action carried out not at all*; 1 = *minimally*; 2 = *to some extent*; 3 = *a good deal*; 4 = *a great deal*). The mean of these is used as the overall Practitioner Score.

This article describes the evaluation of nurses’ competency in delivering the D6 intervention, which was done soon after the end of training, and the assessment of treatment fidelity during the delivery of treatment to participants. The nurse competency sample included one tape recording for each intervention nurse (11 nurses). For the assessment of treatment fidelity two samples were made. The first sample (69 recordings from 21 nurses) was used for quantifying the reliability of the ratings made by the clinical psychologists working on this study. The second sample was larger (266 recordings from 151 patients and 17 nurses) and was used for the fidelity assessment, which was the main focus of this article.

### Nurse competency assessment

The nurse trainer, who was MITI trained, assessed post-training adherence and competency of all nurses in the intervention group using the MITI and BECCI rating scales. One tape recording of a treatment consultation was submitted by each nurse soon after the end of training and then rated on each of the two scales. Nurses were rated as not MI adherent if MITI MI-Adherence was lower than 90% (the “Beginning proficiency” threshold, see Table 1) and MITI Empathy was lower than 3 (which is defined as representing modest success of clinician trying to understand the patient’s perspective [26]). These subscales were chosen because MI-Adherence and Empathy have been shown to be predictive of treatment success [28, 29]. The “Beginning proficiency” and “Competency” thresholds in the MITI manual (Table 1) were considered too high in the context of this study, where consultations included clinical communications that would not be part of a standard MI consultation (for example a physical examination, prescribing, and checking adherence). Any nurses rated as not MI adherent were given extra training and then reassessed. Nurses who were judged to be adherent but who did not meet the higher MITI threshold levels were expected to continue to improve with extra supervision.

### Sampling for inter-rater reliability assessment

A researcher assessed every tape recording and removed duplicates and recordings where session number could not be identified. Of the tape recordings that were from treatment sessions two to four, and where there was a recording of a treatment session that lasted 20 min or more, stratified probability sampling was used to select three recordings from each nurse. Within each nurse stratum, the first tape recording was chosen at random and the second recording was then chosen at random after removing recordings from the previously-selected individual and session from the sample set. The same technique was used to sample the third recording.

The sample comprised 69 tape recordings (representing 3.4% of the total number of all treatment sessions, and 4.0% of sessions where a recording had been made). A 20-min window in the middle of the recording was rated using the MITI (by raters A and B). Of this sample, 32 recordings were rated using the BECCI by raters B and C. Recordings in this sub-sample featured in both the reliability assessment and fidelity assessment (described in next section). Rater C listened to and coded a 20-min window in the middle of the recording whilst rater B assessed the entire recording (raters B and C’s assessments were originally intended for different purposes). Raters received suitable training for whichever scale they used and were blind to treatment allocation. This sample was used in order to check the inter-rater reliability of raters who assessed recordings in the fidelity study.

### Sampling for fidelity assessment

The sampling procedure for the fidelity assessment selected tape recordings from participants who had at least one recording from sessions two, three, and four, and where treatment centre was identifiable (there was no minimum duration of session length). This set included 353 recordings from 154 participants (31 participants with one recording; 47 with two; and 76 with three). Random sampling stratified by participant was used to select two recordings from each of the participants with all three recordings. If only one or two recordings were available for a given participant then these were chosen for subsequent fidelity assessment.

The sample included 266 usable tape recordings (127 recordings in intervention arm) from 17 nurses’ consultations with 151 participants and 11 recordings where the conversation could not be heard. The usable recordings represented 13.1% of all treatment sessions and 15.4% of sessions where a recording was made. The whole duration of each recording was rated using the MITI (rater A) and BECCI (rater B). Raters were blind to treatment allocation.

### Data analysis

Statistical analyses were conducted using Stata version 14. In order to assess inter-rater reliability for the MITI global scores and BECCI Practitioner Scores, intra-class correlation coefficients (ICCs) were estimated using a mixed model. The model included a fixed effect for rater, a random effect for tape recording, and a random effect for primary care nurse in order to account for clustering. It assessed consistency between individual ratings by estimating ICCs at the participant-within-nurse level. The MITI global scores and BECCI Practitioner Score were summarized within the intervention arm shortly after the end of training and during delivery of intervention. Mixed effects regression models with random effects for primary care nurse and participant or Somers’ D tests with sampling from the highest level of the cluster structure (i.e. primary care nurse) were used to compare the fidelity of the psychological therapy delivery between participants in the two trial arms.

## Results

### Nurse and participant sample characteristics

Twenty-three primary care nurses participated in the trial, with 11 randomised to the intervention arm, and 12 to control. They were all female, with a mean age of 48 (SD 8.5) years. Fourteen (61%) of the nurses were white, six (26%) black, and 3 (13%) Asian or other ethnicity.

In terms of previous training in psychological therapies, nine had no previous experience (4 intervention, 5 control), two had completed a module as part of a degree course (1 intervention, 1 control), two had completed some training in MI as part of a smoking cessation course (1 intervention, 1 control), two had undertaken one day or less of MI training (1 intervention, 1 control), one had completed some MI training as part of the Co-Creating Health Programme (intervention), and one had some experience as part of a nursing qualification (intervention). Data on previous training were not available for six nurses.

The participant sample from which the tape recordings were drawn (treatment fidelity assessment sample) included 151 adults with T2D (45% of the total number of participants who entered into the trial), of whom 74 (49%) were in the psychological treatment trial arm. Mean age was 59.4 (SD 11.1) years and 77 (51%) were female. Sixty-eight (45%) were white, 60 (40%) black, 13 (9%) Asian, and 10 (7%) of another ethnicity. Median duration of diabetes was 9 (IQR 6–13) years and mean pre-intervention glycated haemoglobin was 80.1 mmol/mol (SD 18.9) or 9.5% (SD 1.7).

### Nurse competency

The nurse trainer assessed post-training treatment adherence and competency using the MITI and BECCI rating scales. One nurse was not considered MI adherent post training (using MITI MI-Adherence and Empathy subscales) and therefore was given extra training by the clinical psychologist. Upon reassessment she was deemed MI adherent in the therapy. Mean MITI and BECCI competency scores post-training are presented in Table 2.

**Table 2:** Competency scores assessed post-training

Domain	Post-training
MITI Global Spirit (mean; SD)	3.42 (0.67)
MITI Global Empathy (mean; SD)	4.09 (1.04)
Reflection-to-Question Ratio (median; IQR)	0.67 (0.45–0.82)
Percent Open Questions (median; IQR)	45.5 (25.0–72.2)
Percent Complex Reflections (median; IQR)	9.1 (0–28.6)
Percent MI-Adherent (median; IQR)	86.2 (76.9–100)
BECCI Practitioner Score (mean; SD)	2.78 (0.50)

### Inter-rater reliability

Estimates of intraclass correlation coefficients for the global MITI scores and BECCI Practitioner Score are reported in Table 3. These estimates suggested that inter-rater reliability was good (between 0.60 and 0.74) or excellent (> 0.75) for both scales, according to previously defined thresholds [30]. Reliability was greater for MITI, where all ratings were for the 20-min section in the middle of each recording, compared to BECCI, where one coder rated 20-min windows and another rated the full duration of recordings.

**Table 3:** Intraclass correlation coefficients for MITI global scores and BECCI Practitioner Score

Domain	ICC	95% confidence interval
MITI Global Spirit	0.89	0.83–0.93
MITI Global Empathy	0.91	0.86–0.94
BECCI Practitioner Score	0.71	0.52–0.85

### Fidelity assessment

MITI domain scores summarised by trial arm along with the results of the mixed model or Somers’ D tests comparing trial arms are given in Table 4. Estimated standardised mean differences for the MITI global scores were 1.11 (Spirit) and 0.83 (Empathy). There was strong evidence of group differences in favour of the intervention for the global scores of Spirit and Empathy, the percentage of questions that were open, and of percentage of sessions that were MI adherent. There was no evidence of a group difference in percentage of reflections that were complex or the reflection-to-question ratio.

**Table 4:** MITI summary scores during treatment delivery by treatment allocation group

MITI Domain	Attention control group (mean; SD)	Intervention group (mean; SD)	z-test (from mixed model)	95% confidence interval for mean difference
Global Spirit	2.63 (1.12)	4.03 (1.05)	*z* = 4.50, *p* < .001	0.81, 2.06
Global Empathy	3.40 (0.98)	4.23 (0.89)	*z* = 4.55; *p* < .001	0.49, 1.23
	Attention control group (median; IQR)	Intervention group (median; IQR)	z-test (from Somers’ D)	
Reflection-to-Question Ratio	0.50 (0.33–0.71)	0.44 (0.32–0.61)	*z* = −0.55; *p* = .58	
Percent Open Questions	23.1 (13.3–37.5)	46.5 (33.3–57.1)	*z* = 4.17, *p* < .001	
Percent Complex Reflections	55.6 (41.9–71.4)	53.8 (40.0–71.4)	*z* = 0.12, *p* = .90	
Percent MI-Adherent	21.4 (10.0–35.0)	63.4 (33.3–83.3)	*z* = 3.68; *p* < .001	

Numbers and proportions of sessions in the intervention arm that were rated as above MITI’s “Beginning proficiency” and “Competency” thresholds for each domain are summarised in Table 5 [26]. This table summarises how many treatment sessions were assessed as meeting these thresholds within each of the trial arms.

**Table 5:** Numbers and proportions of sessions rated as above MITI’s “Beginning proficiency” and “Competency” thresholds for domains by treatment allocation group

MITI Domain	“Beginning proficiency”		“Competency”	
	Attention control group (n; %)	Intervention group (n; %)	Attention control group (n; %)	Intervention group (n; %)
Global Spirit	34 (24.5)	92 (72.4)	30 (21.6)	88 (69.3)
Global Empathy	71 (51.1)	103 (81.1)	71 (51.1)	103 (81.1)
Reflection-to-Question Ratio	17 (12.2)	9 (7.1)	4 (2.9)	0 (0)
Percent Open Questions	13 (9.4)	54 (42.5)	5 (3.6)	9 (7.1)
Percent Complex Reflections	106 (76.3)	98 (77.2)	87 (62.6)	78 (61.4)
Percent MI-Adherent	1 (0.7)	26 (20.5)	1 (0.7)	25 (19.7)

Mean BECCI Practitioner Score in the control arm was 1.07 (SD 0.48) and in the intervention arm was 1.42 (SD 0.51). A z-test from a mixed effects model showed a significant difference in the BECCI Practitioner Scores between the treatment arms (z = 3.22, *p* < .01, 95% CI 0.15–0.62). The estimated standardised mean difference was 0.75.

## Discussion

This article describes the assessment of the delivery of a nurse-led psychological therapy in the context of a cluster RCT aimed at improving persistent suboptimal glycaemic control in people with T2D. Treatment fidelity and contamination were evaluated by comparing the levels of MI and CBT skills in the two trial arms. At the end of training, nurses in the intervention group were considered competent in D6 skills at a basic level (according to “Beginning proficiency” thresholds) and it appears that there was improvement in some MI skills during delivery of the intervention. For example, MITI Global Spirit and the proportion of reflections that were complex improved. The active intervention delivered to trial participants was statistically superior in Spirit and Empathy, open questions, MI-Adherence, and behaviour change scores compared to attention control. There were no group differences in the proportion of complex reflections or the reflection-to-question ratio. In clinical terms the differences between the trial arms were smaller than expected. The levels of treatment fidelity suggested that some participants in the psychotherapy arm did not receive high fidelity treatment, whilst some in the attention control arm received aspects of the psychological intervention.

In the active intervention arm, findings were partly consistent with the practice of MI, where the clinician collaborates with, supports, and allows the patient to take control of the need for change by listening empathically and using open-ended questions. This was demonstrated by high levels of Spirit and Empathy and a clear majority of treatment sessions being MI-Adherent. The superiority of MI-Adherence and Empathy when comparing the trial arms was particularly important as these have been shown to be predictive of treatment success [28, 29]. However, there were some challenges in providing high fidelity psychotherapy. Specifically, approximately only half of reflections were complex, a similar proportion of questions were open, the ratio of reflections to questions was slightly lower in the intervention group compared to control, and the level of achieved behaviour change fidelity (from the BECCI) was rated between “minimal” and “to some extent”.

There were a number of possible reasons why nurses may not have exceeded MITI’s “Beginning proficiency” levels. The most apparent of these is that the nurses did not self-select to take part in D6. All primary care surgeries meeting the eligibility criteria in the five boroughs were invited to participate. Of those that agreed, the surgery allocated a nurse to take part in the study. Some nurses were more enthusiastic about their participation than others. It is also possible that the skills that showed the lower fidelity levels reflected particular aspects of MI or CBT that are difficult to teach to clinicians who are not specialists in psychological treatment. An interview study with the nurses suggested that not all may be suited to the acquisition of psychological skills [31]. For example, nurses expressed concern about over-stepping their professional roles, feeling that it was inappropriate for them to deliver specialist psychological intervention and described feeling under pressure to participate in the research. Some felt undersupported by their primary care surgery and others resented the extra workload as a result of participating in the trial. Although the surgeries were remunerated for participation, the trial did not provide direct individual financial compensation. One solution to this problem may be to assess inherent competencies prior to training, enabling a process of selection whereby the most suitable nurses are recruited. This is a similar idea to that put forward in an assessment of treatment fidelity of nurse-led MI in pain rehabilitation, where the authors suggested that more rigour was necessary in the selection of MI counsellors [32]. It is not currently possible to distinguish whether D6 nurses possessed existing psychological skills, which were not especially built upon, or whether they learned skills to a basic level but then failed to improve materially upon them.

In the attention control arm, the moderate levels of Spirit and Empathy of MI, the ratio of reflections to questions, which was slightly higher than in the psychological treatment arm, and the fact that just over half of reflections were complex suggested that there was some delivery of MI. On the other hand, the behavioural change index summary score was low in this trial arm. The evidence of delivery of active intervention in the control arm was surprising given the design of the trial. Specifically, cluster randomisation was used in part to avoid a given clinician being trained in the delivery of psychological treatment and then introducing elements of this to participants in the attention control arm. The contamination that took place despite this design may have been due to a number of reasons. For instance, some primary care nurses already possessed skills that were consistent with psychological treatment. Two control nurses are known to have had experience of MI before the trial: one had received brief training in it and one had applied it to smoking cessation. Other reasons include the impact of giving extra time to deliver standard care as part of the attention control design; finally, participation in the trial may itself have induced nurses to provide a slightly different type of standard care.

The primary analysis of D6 included a fidelity assessment of a small sample of therapy session recordings (*n* = 69) in both treatment groups, using both the MITI and the BECCI [7]. The researchers sampled three tape recordings from each nurse and rated only a 20-min window in the middle of each recording. Those findings showed a similar trend to those reported here, but the trial arm differences were estimated to be smaller and had larger standard errors. We consider that, despite the labour-intensive nature of the fuller assessment and the increased costs of employing trained raters (usually psychologists), it is worth rating treatment fidelity for participants (ideally a large sample or all of them) rather than clinicians in order to generate more representative observations of treatment receipt in a trial. Costs may come down with developments in machine learning and automated fidelity evaluation.

In summary, the results indicate that the intervention did not represent the highest level of psychotherapy fidelity, whilst those allocated to receive attention control appeared to receive some components of the intervention. The findings suggest that a large estimate of effectiveness of the intervention, where comparison groups are defined by treatment offer, may be unlikely. There may be utility in an efficacy analysis which estimates treatment effect amongst a sub-population who would receive either high fidelity psychological treatment or pure attention control if offered.

## Conclusions

There were many factors that may have contributed to limited development in skills, including individual nurse characteristics and organisational factors such as lack of support and appropriate surgery infrastructure [31]. Future studies should focus on selection strategies for nurses that maximise chances of success, enhance the training of nurses, consider comparing the comparator treatments of standard care and attention control, or consider the possibility that primary care nurse acquisition of high-level MI and CBT skills is not a viable approach to improved self-management among diabetic patients with persistent suboptimal control. Similar RCTs should assess treatment fidelity in a large sample of participants and should evaluate both treatment receipt in the intervention arm and the absence of intervention in the control arm. This enables an assessment of what treatments participants received and allows researchers to account for this in an efficacy analysis.

Primary care nurses struggled to acquire and deliver psychological skills such as MI and CBT to a high level, despite the use of an intensive, manualised training programme with ongoing supervision by an experienced clinical psychologist. Further studies may be needed to determine whether, for patients to benefit from such therapies, a different skill set may be needed in the healthcare professional or a re-organisation of nurse practitioner time to allow for greater engagement in training and delivery.

## Abbreviations

BECCI: Behaviour Change Counselling Index
CBT: Cognitive behavioural therapy
D6: Diabetes 6
ICC: Intraclass correlation coefficient
MI: Motivational interviewing
MITI: Motivational Interviewing Treatment Integrity
RCT: Randomised controlled trial
T2D: Type 2 diabetes

## References

[1] Craig P, Dieppe P, Macintyre S, Michie S, Nazareth I, Petticrew M (2013). Developing and evaluating complex interventions: the new Medical Research Council guidance. Int J Nurs Stud.

[2] Moore G, Audrey S, Barker M, Bond L, Bonell C, Hardeman W, Moore L, O’Cathain A, Tinati T, Wight D, Baird J (2015). Process evaluation of complex interventions: Medical Research Council guidance. Br Med J.

[3] Dunn G, Emsley R, Liu HH, Landau S, Green J, White I, Pickles A. Evaluation and validation of social and psychological markers in randomised trials of complex interventions in mental health: a methodological research programme. Health Technol Assess. 2015;19(93) 10.3310/hta19930 PMC4781463 26560448

[4] Moore G, Audrey S, Barker M, Bond L, Bonell C, Hardeman W, Richards DA, Hallberg IR (2015). Process evaluation of complex interventions: a summary of Medical Research Council guidance. Complex Interventions in Health: An overview of research methods.

[5] Kohrt B, Ramaiya M, Rai S, Bhardwaj A, Jordans MD (2015). Development of a scoring system for non-specialist ratings of clinical competence in global mental health: a qualitative process evaluation of the enhancing assessment of common therapeutic factors (ENACT) scale. Glob Ment Health.

[6] Torgerson DJ (2001). Contamination in trials: is cluster randomisation the answer?. Br Med J.

[7] Ismail K, Winkley K, de Zoysa N, Patel A, Heslin M, Graves H, Thomas S, et al. Cluster randomised controlled trial of a nurse-led psychological intervention for type 2 diabetes: Diabetes-6 study. Br J Gen Pract. In press. 10.3399/bjgp18X696185 PMC6058638 30012812

[8] King P, Peacock I, Donnelly R (1999). The UK prospective diabetes study (UKPDS): clinical and therapeutic implications for type 2 diabetes. Br J Clin Pharmacol.

[9] Gæde P, Lund-Andersen H, Parving H-H, Pedersen O (2008). Effect of a multifactorial intervention on mortality in type 2 diabetes. N Engl J Med.

[10] Davies MJ, Heller S, Skinner TC, Campbell MJ, Carey ME, Cradock S (2008). Effectiveness of the diabetes education and self management for ongoing and newly diagnosed (DESMOND) programme for people with newly diagnosed type 2 diabetes: cluster randomised controlled trial. Br Med J.

[11] Ciechanowski PS, Katon WJ, Russo JE (2000). Depression and diabetes: impact of depressive symptoms on adherence, function, and costs. Arch Intern Med.

[12] Peyrot M, Rubin RR, Lauritzen T, Snoek FJ, Matthews DR, Skovlund SE (2005). Psychosocial problems and barriers to improved diabetes management: results of the cross-National Diabetes Attitudes, wishes and needs (DAWN) study. Diabetic Med.

[13] National Institute for Clinical Excellence. Type 2 diabetes in adults: management. NICE Guidelines 2015. 26741015

[14] Alam R, Sturt J, Lall R, Winkley K (2009). An updated meta-analysis to assess the effectiveness of psychological interventions delivered by psychological specialists and generalist clinicians on glycaemic control and on psychological status. Patient Educ Couns.

[15] Ismail K, Thomas SM, Maissi E, Chalder T, Schmidt U, Bartlett J, Patel A (2008). Motivational enhancement therapy with and without cognitive behavior therapy to treat type 1 diabetes: a randomized trial. Ann Intern Med.

[16] Hardeman W, Lamming L, Kellar I, De Simoni A, Graffy J, Boase S, Sutton S (2014). Implementation of a nurse-led behaviour change intervention to support medication taking in type 2 diabetes: beyond hypothesised active ingredients (SAMS consultation study). Implement Sci.

[17] Jansink R, Braspenning J, Laurant M, Keizer E, Elwyn G, van der Weijden T, Grol R (2013). Minimal improvement of nurses’ motivational interviewing skills in routine diabetes care one year after training: a cluster randomized trial. BMC Fam Pract.

[18] Arkowitz HW, Westra HA (2004). Integrating motivational interviewing and cognitive Behavioural therapy in the treatment of depression and anxiety. J Cogn Psychother.

[19] Miller WR, Rollnick S (2002). Motivational interviewing: preparing people for change.

[20] Whittemore R, Melkus GD, Sullivan A, Grey M (2004). A nurse-coaching intervention for women with type 2 diabetes. Diabetes Educ.

[21] Ismail K, Winkley K, Rabe-Hesketh S (2004). Systematic review and meta-analysis of randomised controlled trials of psychological interventions to improve glycaemic control in patients with type 2 diabetes. Lancet.

[22] Madson MB, Campbell TC (2006). Measures of fidelity in motivational enhancement: a systematic review. J Subst Abus Treat.

[23] Rakovshik SG, McManus F (2010). Establishing evidence-based training in cognitive behavioral therapy: a review of current empirical findings and theoretical guidance. Clin Psychol Rev.

[24] Fairburn CG, Cooper Z (2011). Therapist competence, therapy quality, and therapist training. Behav Res and Ther.

[25] Moyers TB, Martin T, Manuel JK, Hendrickson SML, Miller WR (2005). Assessing competence in the use of motivational interviewing. J Subst Abuse Treat.

[26] Moyers T, Martin T, Manuel J, Miller W, Ernst D. Revised global scales: motivational interviewing treatment integrity 3.1. 1 (MITI 3.1. 1). Unpublished manuscript, University of New Mexico, Albuquerque, NM. 2010. https://casaa.unm.edu/download/miti3_1.pdf.

[27] Lane C, Huws-Thomas M, Hood K, Rollnick S, Edwards K, Robling M (2005). Measuring adaptations of motivational interviewing: the development and validation of the behavior change counseling index (BECCI). Patient Educ Couns.

[28] Apodaca TR, Longabaugh R (2009). Mechanisms of change in motivational interviewing: a review and preliminary evaluation of the evidence. Addiction.

[29] Moyers TB, Miller WR (2013). Is low therapist empathy toxic?. Psychol Addict Behav.

[30] Cicchetti DV (1994). Guidelines, criteria, and rules of thumb for evaluating normed and standardized assessment instruments in psychology. Psychol Assess.

[31] Graves H, Garrett C, Amiel SA, Ismail K, Winkley K. Psychological skills training to support diabetes self-management: qualitative assessment of nurses’ experiences. Prim Care Diabetes. 2016:376–82. 10.1016/j.pcd.2016.03.001 27006306

[32] Mertens VC, Forsberg L, Verbunt JA, Smeets RE, Goossens ME (2016). Treatment fidelity of a nurse-led motivational interviewing-based pre-treatment in pain rehabilitation. J Behav Health Serv Res.

